# Case Report: Unveiling the Unseen – Ocular Tuberculosis Presenting as Chalazion

**DOI:** 10.4269/ajtmh.24-0271

**Published:** 2024-08-13

**Authors:** Rucha Karad, Vasireddy Teja, Hardik Patel, Boudhayan Bhattacharjee, Agnibho Mondal, Soumendra Nath Haldar, Bibhuti Saha

**Affiliations:** Department of Infectious Diseases and Advanced Microbiology, School of Tropical Medicine, Kolkata, India

## Abstract

Tuberculosis (TB) is an airborne infectious disease caused by *Mycobacterium tuberculosis* that most commonly affects the lungs. Ocular involvement as part of extrapulmonary TB is noted in around 2–18% of cases of extrapulmonary TB. Any part of the eyes can be affected by the tubercular disease process, and a high index of suspicion is required for accurate diagnosis. Because the location is extrapulmonary, obtaining a proper sample is difficult, and the paucibacillary nature of the disease also makes microbiological detection a diagnostic challenge. Response to antitubercular therapy is usually good, and resolution of clinical features is observed in most cases. Here, we present a case report of a patient presenting with a chalazion-like lesion in the left eyelid that recurred after surgical intervention and did not respond to medical therapy. No history of past TB infection or contact was noted in the patient. An active tubercular lung infection was excluded. On further evaluation, the lesion was microbiologically proven to be of tubercular origin, and the signs and symptoms of the patient completely resolved with proper antitubercular therapy.

## INTRODUCTION

Tuberculosis (TB) is an airborne infectious disease caused by *Mycobacterium tuberculosis.* The respiratory system is the primary site of involvement, but the bacilli can be present in an extrapulmonary site latently and reactivate whenever the immune system of the individual weakens. In 2022, around 10.6 million people suffered from TB worldwide including 5.8 million men, 3.5 million women, and 1.3 million children.^1^ Among extrapulmonary manifestations of TB, orbital TB is a quite uncommon entity. It can affect the eyes and the surrounding orbital tissue. The most common mechanism observed in extrapulmonary involvement is hematogenous spread from the primary lung lesion, but direct inoculation can also lead to localized disease manifestation. The precise incidence of ocular TB is much more difficult to discern, ranging from 1.4% to 18% among the incidence of extrapulmonary TB in various studies.^1^ In ocular TB, most studies showed that uveitis is the common clinical entity associated with the disease, but direct association of the etiology and clinical presentation is difficult to prove.^2^ Another common entity in ocular TB is choroid tubercle, which was demonstrated in 1855 and was visualized with the ophthalmoscope in 1867. In 1883, the causative organism for the condition was proved to be *M. tuberculosis*.^3^ Tuberculosis of the eyelid is an uncommon entity, as its prevalence among ocular TB is very low.

## CASE REPORT

**Figure 1.**
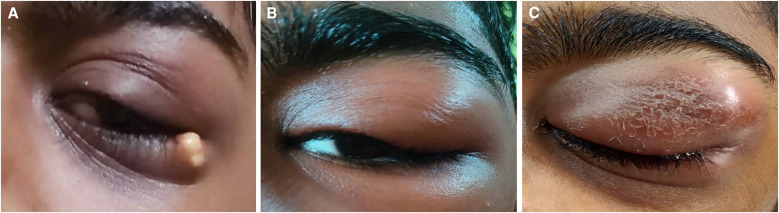
Gradual progression of the eyelid lesion from a pustular lesion (**A**) to involvement of upper and lower eyelids with swelling (**B**) followed by cellulitic changes over upper eyelid (**C**) with visual impairment.

A 16-year-old male resident of Kolkata, with no history of any major illness in the past, presented with left eye swelling for 1 month. The swelling was painful and gradually progressive in nature and started as a pustular lesion over the left lower eyelid. He later developed significant swelling of the involved eye, causing impaired vision within a week of occurrence. He visited an ophthalmologist for the same, and the lesion was drained. Aspirated material, not sent for any microbiology or histopathological investigation as a chalazion, a common eye condition, was the primary suspicion (Figure 1). The patient was given some empirical oral antibiotics, and anti-inflammatory eye drops were also advised. Eye pain and swelling were not completely relieved with this therapeutic intervention. After 10 days, the lesion with orbital swelling reappeared. This time the eye swelling was also associated with a low-grade fever for 10 days.

On examination, the patient was vitally stable. General examination did not reveal any abnormality. There was no lymphadenopathy, organomegaly, or focal neurological abnormality.

The patient's hemoglobin was 11.4 g/dl, total leukocyte count was 7,600 cells/mm^3^, platelet count was 276,000/mm^3^ and his renal function and liver functions tests were within normal limits. His C-reactive protein (CRP) value was 57.2 mg/L, and his erythrocyte sedimentation rate (ESR) was 34 mm/hour. Both the CRP and the ESR were toward the higher side.

Chest X-ray, ultrasonography of the abdomen, and contrast-enhanced computed tomography of the brain with ocular scan did not reveal any abnormalities.

The patient sought infectious diseases consultation in view of nonresolution of his symptoms. A detailed history of the patient, including trauma, exposure to pets, and personal habits, was noted. Considering the endemicity of TB in India, a detailed history was taken regarding past tubercular infection, TB in the family, and contact with TB patients. Aspiration from the lesion was repeated, and the sample was sent for Gram stain, bacterial culture, acid-fast stain, modified acid-fast stain, fungal stain, fungal culture, GeneXpert, and *Mycobacterium* growth indicator tube culture. Histopathological examination was not done as no tissue was taken from the lesion.

Pus aspiration from the lesion showed slender acid-fast bacilli (Supplemental Figure 1), and GeneXpert of aspirated fluid detected *M. tuberculosis*, which was rifampicin sensitive.

Based on the above findings, the patient was diagnosed as having a tubercular eyelid infection, and he was treated with antitubercular therapy including isoniazid (H), rifampicin (R), pyrazinamide (Z), and ethambutol (E) according to the weight-based criteria provided by the National Tuberculosis Elimination Programme. Antitubercular therapy was given for a total duration of 6 months: HRZE for 2 months followed by HRE for 4 months. The patient initially had some gastric intolerance to the therapy, but no other treatment complication was observed.

The patient’s eye swelling started reducing after the fifth day of initiation of antitubercular therapy (Supplemental Figure 2). He was discharged and followed up on an outpatient basis.

## DISCUSSION

Any part, compartment, and tissue of the eyes can be involved in the tubercular infectious process. Eyelid TB is often confused with a chalazion. Because TB is often not included in the differentials, proper evaluation is frequently not undertaken. Most patients are initially treated with oral antibiotics and topical anti-inflammatory agents considering possibility of chalazion; however, there is not enough evidence regarding the therapeutic efficacy of these treatment options in the management of a chalazion.^4^ The actual incidence of eyelid TB is unknown, but according to one retrospective study by Babu et al.^5^ from southern India, about 2% of cases of extrapulmonary TB are due to ocular TB. They found that the most common manifestation of ocular TB was choroidal tuberculoma. However, because this study was done on immunodeficient individuals, the findings may not be generalizable to immunocompetent patients. Only a few case reports of eyelid TB are available,^6^^–^^8^ showing the lack of awareness regarding this uncommon presentation. Among the published literature, one case was presented as a unilateral chalazion similar to our case, but this patient had submandibular lymphadenopathy associated with the eye symptom.^9^ Another case was presented as a mass along the lower eyelid that was later diagnosed as an extra tarsal chalazion of tubercular origin.^10^ Other manifestations of TB in the eyelid are chronic blepharitis or nodules, which may recur after excision. Another case report showed bilateral involvement of the lower eyelid with a chalazion-like presentation in a patient suffering from pulmonary TB.^11^ In our patient there was no history of past infection with TB or TB contact. He also had no symptoms or signs suggestive of active TB infection in other sites. Although the patient had a history of fall while playing in the recent past, contribution of this event to the eye lesion was difficult to confirm.

In such cases, the opinion of an infectious diseases specialist and appropriate sampling allow the early detection of any pathogen.

Tuberculosis should be considered in the differential diagnosis of chronic ocular pathology. Microbiological demonstration of *M. tuberculosis* is important to avoid empirical therapy, as drug-resistant TB is a serious problem that can arise because of improper use of antitubercular therapy. There should be a high index of suspicion for ocular TB not only in endemic areas but also in other parts of the world. Early diagnosis and treatment can avoid progression to impaired vision due to TB.

## Supplemental Materials

10.4269/ajtmh.24-0271 Supplemental Materials
