# Supplementary material for: Case Report: Unveiling the Unseen – Ocular Tuberculosis Presenting as Chalazion
Source: Am J Trop Med Hyg. 2024 Aug 13;111(4):841–3. doi: 10.4269/ajtmh.24-0271 (PMC11448534; doi:10.4269/ajtmh.24-0271)

Supplemental Figure 1. Acid-fast bacteria demonstrated in the aspirated fluid from the eyelid lesion by Ziehl-Neelsen staining.

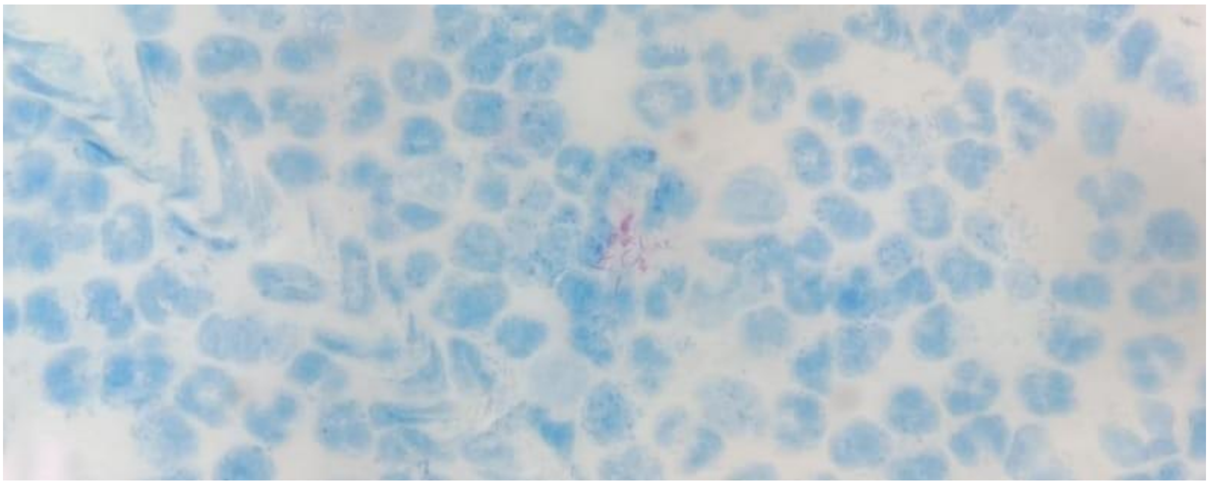

Supplemental Figure 2. Post anti tubercular therapy (6 months) eyelids of the patient, with complete resolution of the swelling.

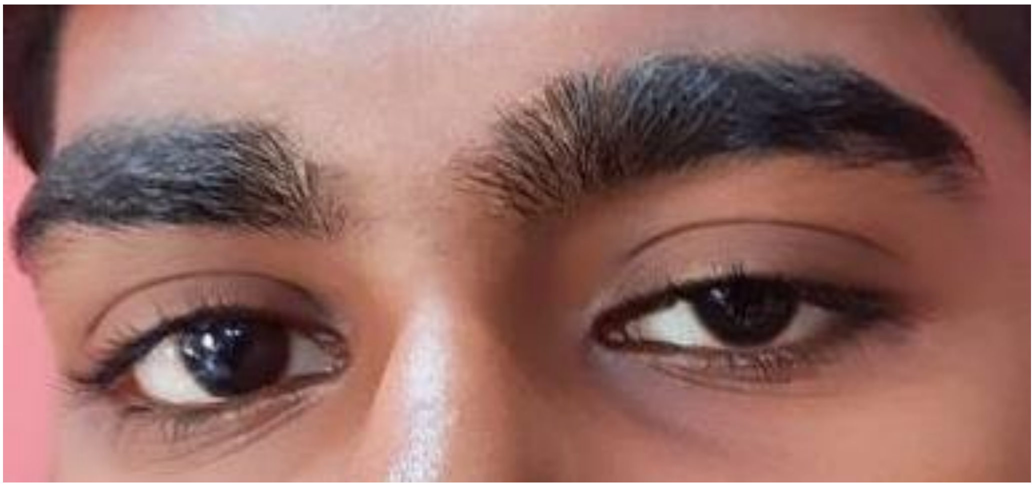

Supplement: Supplemental Materials [file tpmd240271.SD1.pdf]
